# Transanal Endorectal Pull-Through for Hirschsprung’s Disease: Complications and Lessons from Our Practice and the Literature

**DOI:** 10.3390/children11091059

**Published:** 2024-08-29

**Authors:** Andrzej Gołębiewski, Stefan Anzelewicz, Daria Sosińska, Monika Osajca-Kanyion

**Affiliations:** 1 Department of Surgery and Urology for Children and Adolescents, Medical University of Gdansk, 80-210 Gdansk, Poland; stefan.anzelewicz@gumed.edu.pl (S.A.); monika.osajca-kanyion@gumed.edu.pl (M.O.-K.); 2 University Clinical Centre in Gdansk, 80-952 Gdansk, Poland; dsosinska@uck.gda.pl

**Keywords:** Hirschsprung’s disease, transanal endorectal pull-through (TEPT), paediatric surgery, postoperative complications, long-term outcomes

## Abstract

Background/Objectives: Hirschsprung’s disease (HD) is a congenital disorder characterised by the absence of ganglion cells in the distal bowel, resulting in functional obstruction. The transanal endorectal pull-through (TEPT) procedure, a minimally invasive approach, aims to treat HD by removing the aganglionic segment. This study assessed the feasibility, safety, and efficacy of single-stage TEPT in paediatric patients, focusing on postoperative complications, bowel function, and quality of life. Methods: A retrospective cohort study was conducted on 150 children who underwent single-stage TEPT from January 2005 to December 2023 at the Medical University of Gdansk. Data were collected from medical records, including demographics, preoperative assessments, surgical details, postoperative management, and follow-up outcomes. Statistical analyses were performed using Microsoft Excel 365 and the programming language Python 3.12. The mean age at surgery was 13 months, with a male-to-female ratio of 2.75:1. The mean operative time was 129 min, and the mean hospital stay was seven days. Results: Postoperative complications included anastomotic leak (4%), wound infections (15%), and enterocolitis (26%). Redo surgeries were required in 18% of cases due to persistent constipation and obstructive symptoms. This article includes a comprehensive review of the literature. Conclusions: TEPT demonstrates a favourable safety profile and efficacy in treating HD, though significant concerns include complications such as enterocolitis and the need for additional surgeries. Surgical expertise and thorough preoperative and postoperative management are crucial to optimising patient outcomes.

## 1. Introduction

Hirschsprung’s disease (HD) is a rare congenital disorder that affects the development of the enteric nervous system (ENS) in the distal bowel. Typically, neural crest cells migrate from the neural tube to the gut and form the myenteric and submucosal plexuses, which regulate the peristaltic activity of the intestine. In HD, this migration is disrupted, leading to a colon segment lacking ganglion cells and being unable to relax. This causes a functional obstruction and prevents the typical passage of stool, resulting in chronic constipation and bowel distention [1].

The clinical presentation of HD varies depending on the age of the patient. Neonates with HD often have signs such as failure to pass meconium within the first 48 h of life, abdominal bloating, and greenish vomiting. Older children with HD may experience chronic constipation, poor growth, and episodes of enterocolitis [2]. The diagnosis of HD is mainly based on clinical history and a physical examination and confirmed by tests such as a rectal biopsy, which shows the lack of ganglion cells, and contrast enema, which shows a narrowing of the aganglionic segment of the bowel [3]. Surgery is the only definite treatment option for HD.

Orvar Swenson pioneered the first definitive surgical treatment for HD in 1948 by completely resecting the aganglionic segment via an abdominal incision and anastomosing the normal bowel to the anus. Subsequent surgical innovations included the Duhamel procedure (1956, side-to-side anastomosis between the ganglionic bowel and the aganglionic bowel), which bypassed the aganglionic segment through a retrorectal approach, and the Soave procedure (1964, removal of the diseased bowel while preserving the rectal muscular layer), which preserved the anal canal’s integrity by performing a submucosal dissection. In 1995, Georgeson et al. [3] introduced laparoscopy as an aid in HD surgery.

The transanal endorectal pull-through (TEPT) procedure has emerged as a prominent surgical technique in treating Hirschsprung’s disease (HD), particularly in paediatric patients. Initially described by de la Torre-Mondragón and Ortega-Salgado in 1998 [4], TEPT has gained widespread acceptance due to its minimally invasive nature (a single-stage procedure that avoids an abdominal scar [5]), and the potential for reduced postoperative morbidity compared to traditional open surgical methods [2]. TEPT is, at present, the most popular [6] and a procedure of choice for many institutions [7]. Despite its advantages, the technique has limitations and complications, necessitating a thorough evaluation and comparison with other established surgical approaches.

Some patients with Hirschsprung’s disease require more than one surgery to achieve a satisfactory functional outcome. This may be due to the presence of long-segment disease, complications of the primary pull-through procedure, and other factors like pelvic-floor insufficiency [8], or internal anal sphincter achalasia [9]. Additional surgeries may include diverting ostomies, emergency surgeries, stoma closures, and redo surgeries; multiple surgeries can increase the risk of short bowel syndrome and malabsorption. 

Since 2004, the Department of Surgery and Urology for Children and Adolescents, Medical University of Gdansk, has adopted the transanal endorectal pull-through (TEPT) procedure [10]. While this type of surgery is usually effective, various complications can arise, including early (anastomotic leak, infection, bleeding, and enterocolitis), late (strictures, constipation/incontinence, and obstruction), and psychosocial issues.

## 2. Materials and Methods

This is a retrospective cohort study. We have performed a comprehensive retrospective analysis of the transanal endorectal pull-through (TEPT) procedure performed in our department from January 2005 to December 2023. This study’s objectives were to evaluate the feasibility, safety, and efficacy of single-stage transanal endorectal pull-through in children with Hirschsprung’s disease and to assess short-term and long-term outcomes, including surgical complications, gastrointestinal function, and overall quality of life.

Medical records of all eligible patients from hospital archives and electronic databases were retrieved, and relevant data were extracted using a standardised data collection form. Data entries were verified and cross-checked for accuracy. Table 1 presents the inclusion and exclusion criteria, and Table 2 summarises the data collected in this study. 

The results were statistically analysed in Microsoft Excel 365 using the programming language Python 3.12 (Python Software Foundation, 2023). We utilised the GPT-4o model of ChatGPT (OpenAI, San Francisco, CA, USA, 2023) to provide statistical analysis support.

## 3. Results

### 3.1. Demographics

During this study period, we operated on 150 patients who met the inclusion and exclusion criteria. The mean age at surgery was 13 months (±10.21 months). However, the K-means clustering method using Python revealed two subgroups: 128 infants with a mean age of ~6.5 months (±2.1 months) and 22 older children with a mean age of ~2.3 years (±1.2 years).

The gender distribution analysis revealed 110 male and 40 female patients (male-to-female ratio of 2.75:1). The gender bias is consistent with the epidemiology of the condition; there is a well-documented male preponderance [8].

The mean weight for the infants’ group was 6.0 kg (±0.6 kg), and the mean for the older children was 10.5 kg (±0.9 kg).

Trisomy 21 was present in 21 cases (14%). A minority of patients had additional comorbidities, including hypospadias, gastroesophageal reflux, small bowel atresia, duodenal atresia, gastroschisis, undescended testes, Haddad syndrome (2 cases), and Mowat–Wilson syndrome (1 case).

### 3.2. Preoperative Assessment

The most common symptoms in the infant group included chronic constipation, abdominal distention, poor feeding, and vomiting. In the older children’s group, the most common symptoms included constipation, distention, failure to thrive, and foul-smelling stools. Eight children in the infant group presented with bilious vomiting (6.3%); this symptom was not seen in the older age group. Enterocolitis was present before surgery in 10 children in the infant group (7.8%) and 4 children in the older group (18%).

All patients had contrast enemas performed (barium enema) and rectal biopsy (infant group: mostly suction rectal biopsy, older children: full thickness rectal biopsy) confirming histopathologic changes typical for HD. Fifteen patients needed to have the biopsy repeated (10%). A total of 64% of patients had anorectal manometry performed as a preoperative workup. Additionally, 25% of our patients underwent colonic mapping: 0% laparoscopically and 70% via laparotomy. 

Patients undergoing surgical treatment for Hirschsprung’s disease (HD) underwent a multifaceted preparation process encompassing bowel management, dietary adjustments, and antibiotic prophylaxis (neonates: ampicillin and gentamicin; infants and older children: cefazoline and metronidazole).

### 3.3. Surgical Details

All patients underwent primary transanal endorectal pull-through. Thirty-three (22%) patients needed additional laparoscopic (18%) or laparotomy (4%) assistance during surgery. Intraoperative frozen section biopsies were inconclusive in 14% of cases. The mean operative time was 129 min ± 15.2 min. The mean operative time differed non-significantly (infant group 128 ± 14.2 min; older children 132 ± 20 min; *p*-value 0.3768 in *t*-test). Intraoperative complications included in the infants’ group were as follows: bleeding, urethral injury (1 case), twisted pull-through (2 cases), suspected anal sphincter damage (3 cases) due to prolonged anal stretching, and need for ICU admission (6 cases). In older children, only bleeding was observed as a main intraoperative complication.

The resected bowel length in the infant’s group was 22 cm (range 15–30 cm); in older children, it was 25 cm (range 15–35 cm). The difference was significant in the Independent Samples *t*-test (*p*-value 0.0126). 

### 3.4. Postoperative Management

The mean hospital stay was seven days (5–23 days). Postoperative antibiotic therapy was continued during the stay (the antibiotics administered were the same as those used for prophylaxis). Four children needed a blood transfusion due to postoperative rectal bleeding. Loose stools were commonly observed after surgery; perineal excoriations were observed in 20% of the patients. Wound infections occurred in 15% of the patients and were managed conservatively. In six cases, anastomotic leak was observed (4%), treated by bowel rest and percutaneous drainage in two cases alone and in four instances necessitating diverting colostomy. Sixteen children needed rectal irrigations after the surgery.

### 3.5. Follow-Up

The minimal follow-up time was six months. One patient needed urethral dilation after intraoperative damage to the urethra. Twenty-seven patients (18%) needed redo surgery (persistent constipation, transition zone pull-through, aganglionic bowel pull-through, bowel obstruction and twisted pull-through segment). In 21 patients, anastomotic strictures were observed despite postoperative routine Hegar dilations. The strictures were managed by surgical revision, anal dilations with dilators, or balloon dilations combined with steroid (triamcinolone) injections. Hirschsprung-associated enterocolitis was observed in 40 patients (26%). Ten patients from the older age group (25%) received prophylactic oral metronidazole for five days each month. Redo surgery was performed on five patients. In the rest of the patients, the episode was seen only once. Soiling and pseudo-incontinence were observed in 18% of the patients, and obstructive symptoms were seen in 30% of the patients, often accompanied by soiling.

## 4. Discussion and Review

The transanal endorectal pull-through (TEPT, TERPT, or ERPT) procedure is a prominent surgical technique for treating Hirschsprung’s disease (HD), offering a minimally invasive approach to remove the aganglionic segments of the bowel, with the primary goal of preservation of the internal sphincter muscle. This review aimed to highlight the limitations, possible complications and overall effectiveness of TEPT, enabling comparison with other methods, such as laparotomy or laparoscopy-assisted colonic mobilisation (LEPT) or the traditional Duhamel, Soave, and Swenson procedures.

### 4.1. TEPT Innovations

The transanal endorectal pull-through procedure has undergone significant evolution since its inception. Early surgical approaches to HD, such as the Swenson, Duhamel, and Soave procedures, while effective, involved large abdominal incisions and carried moderate-to-high risks to pelvic structures [4]. In 1995, Georgeson et al. [3] introduced laparoscopy as an aid in HD surgery, initially using it to identify and biopsy the transition zone and mobilise the rectum and sigmoid colon. This laparoscopic assistance, although not entirely transanal, paved the way for further minimising invasiveness in the field [11]. TEPT has evolved over the years to become a preferred approach due to its reduced invasiveness and improved outcomes compared to traditional methods [12]. Some modifications of the technique involve the use of frozen section studies of the transition zone [7], laparoscopic assistance [11], cuff length and anastomosis modifications [13], utilisation of the NOTES concept and indocyanine green fluorescence imaging [14], and the introduction of Enhanced Recovery After Surgery protocols [15].

### 4.2. TEPT Limitations

Limitations of the transanal endorectal pull-through (TEPT) procedure have been identified through various studies. One significant limitation is the incidence of postoperative complications such as enterocolitis, which can be a severe and potentially life-threatening complication associated with Hirschsprung’s disease (HD) surgeries [16]. While the overall incidence of enterocolitis may be low, the TEPT technique has been associated with a higher risk of enterocolitis than other procedures like the Duhamel technique. Additionally, the presence of diarrhoea, which is a common symptom of Hirschsprung-associated enterocolitis, can occur in a significant percentage of patients postoperatively [16]. 

Another limitation of TEPT is the variability in outcomes related to bowel function, with reports of constipation rates ranging widely from 3% to 28% [17]. This variability in postoperative bowel function outcomes underscores the challenges in achieving consistent results with TEPT. Furthermore, reports indicate that constipation and obstructive syndromes can persist or even worsen after the pull-through procedure in some cases [18].

Evaluating quality of life (QoL) and faecal continence following TEPT has also been a concern. Studies have highlighted the use of various questionnaires to assess QoL and faecal continence, but the interpretation of results may be limited by the lack of standardised cuff length data and inconsistent reporting on the advantages and disadvantages of different cuff lengths [5,18].

Another concern is the limited visualisation and accessibility, particularly in cases involving long segmental or total colonic aganglionosis. Secondly, the transanal approach makes it more challenging to mobilise the bowel adequately, potentially leading to incomplete resection of aganglionic segments. This may lead to the need for reoperation, as seen in our experience. It seems less prevalent in open or laparoscopy-assisted procedures, which provide better exposure. 

However, bias due to retrospective analysis is one of this study’s limitations [19]. So far, there is insufficient information regarding the long-term outcomes of transanal endorectal pull-through. Specifically, few reports utilise validated questionnaires to assess these outcomes [20]. Follow-up duration and different surgeons’ expertise in prospective analyses also vary, making it difficult to draw definitive conclusions.

### 4.3. TEPT Complications

Potential postoperative complications include infection at the surgical site, bleeding within the abdomen, perforation of the intestines, bowel obstruction, or formation of an abnormal connection such as rectovesical or rectovaginal fistulas, anastomotic leakage, and inflammation. Those complications are no different from ones caused by other procedures performed on the distal gut. Over the long term, patients may experience occasional leakage of stool, incontinence, narrowing of the intestines, obstruction, and enterocolitis. In our clinic, complications started to decrease as more procedures were performed, showing the importance of the surgeon’s expertise. 

Based on the clinical experience of our institution with a cohort of 150 patients, all individuals exhibited an increased frequency of loose stools, which predominantly normalised within 3 to 6 months postoperatively. However, eight patients presented with postoperative complications, including episodes of constipation, subacute intestinal obstruction, and enterocolitis. These complications were attributed to the incomplete resection of aganglionic bowel segments due to inaccurate intraoperative histopathological assessments, necessitating repeated transanal endorectal pull-through (TEPT) surgeries. Our analysis identified additional complications, including mucosal prolapse, intestinal anastomotic stricture leading to impaired stool passage, faecal soiling, incisional hernia, isolated urethral stricture, and neurogenic bladder instances.

#### 4.3.1. Faecal Incontinence

Faecal incontinence has been mentioned as one of the leading complications of TEPT surgery in the literature, yet the frequency varies widely across different studies. For instance, in a retrospective population-based study involving 103 patients with rectosigmoid aganglionosis who underwent TEPT, 29% experienced episodes of faecal incontinence occurring more than once a week for many years post-surgery [21], while in a different study of 281 patients post endorectal pull-through surgery, 12% reported experiencing such symptoms [2]. Another meta-analysis indicated that faecal incontinence incidents happening at least once a week were observed in 54% of the patients [22]. 

#### 4.3.2. Defecation Patterns 

Persistent abnormal defecation frequency and related social issues were also reported and claimed to show no improvement in one of the extensive studies [6]. However, numerous reports have suggested that after undergoing the pull-through procedure, defecation patterns tend to be better as the patient grows older [23]. 

Obstructive symptoms have been described to occur in 6% to 40% of the patients who underwent surgical management for HD [24]. Constipation was reported in 25% of patients, which was also correlated with higher squeeze pressure in manometry [22]. Another meta-analysis reported constipation in 9% of 146 patients in a complete follow-up [21]. However, in a different study, constipation occurred in 42% of cases, and most of the cases were mild [20].

#### 4.3.3. Faecal Soiling 

In one of the extensive analyses, faecal soiling incidence was higher in children with an associated syndrome or other illnesses in an average of 62% of the patients, who also reported unchanged occurrence of the symptoms between check-ups [25]. However, another study reported this symptom in only 11% of the patients in a follow-up [16]. 

#### 4.3.4. Hirschsprung-Associated Enterocolitis (HAEC) 

In a large population study of 200 children, postoperative HAEC affected approximately one-third of the patients [26]. Another study showed a rate of 10.2% of 899 patients postoperatively, while recurring enterocolitis was reported in 2% of children [17]. The latter form of enterocolitis is also claimed to occur more often among children with associated illness [25]. A systemic literature-based search, including 9744 preoperative and 8568 postoperative patients, showed that the pooled prevalence for HAEC was 18.2% [27]. 

Anastomotic strictures were reported in 8% of 899 children who underwent the TEPT procedure, increasing the risk of HAEC [17]. Anastomotic leak, however, was reported among 1.5% of the patients [26]. 

#### 4.3.5. Urological Complications 

Urological complications are poorly documented in the literature. They appear to be more prevalent in children with concomitant genetic syndromes [28] and are often associated with worse bowel outcomes [29]. A potential concern of pelvic dissection is the damage to the pelvic splanchnic nerves, which may impair bladder emptying, urinary continence, and sexual function. However, evidence suggests that TEPT preserves the integrity and functionality of the genitourinary tract in the long term [30].

#### 4.3.6. Complications in Similar Retrospective Studies

Table 3 presents the results of studies comparable to our retrospective study. The study by Samujh et al. [31] is a retrospective review of 71 children after TEPT. A study by Szymczak et al. [32] provides data on complications following TEPT surgery for HD, including a retrospective review of 38 patients. Prytula et al. [33] performed a long-term follow-up of 209 patients after TEPT, including a comparison between those with and without laparoscopic assistance. Beltman et al. [34] examined 106 patients who underwent TEPT, focusing on an analysis of postoperative complications.

### 4.4. TEPT and Other Surgical Procedures

Several surgical techniques, such as TEPT and the Soave, Svenson and Duhamel techniques, are available for treating Hirschsprung’s disease. The comparative outcomes of these procedures regarding bowel function are generally favourable, but some differences have been reported. For instance, the Duhamel procedure was associated with higher rates of constipation and reduced faecal awareness than those of TEPT, and these effects persisted into adulthood. Conversely, TEPT had a higher risk of postoperative enterocolitis and anastomotic stricture than that of the Duhamel procedure [29].

A meta-analysis of 315 patients showed that the postoperative enterocolitis rate was 21.3% for TEPT and 5.7% for the Duhamel pull-through [6]. Five studies in the meta-analysis reported anastomotic stricture only after TEPT, affecting 11% of the patients, while none of the 175 patients who underwent the Duhamel pull-through had this complication. Four studies with 164 patients reported anastomotic leaks only after the Duhamel pull-through in 3 of 104 patients and no cases after TEPT. Another analysis indicated that faecal incontinence was similar between the two procedures, but the Duhamel pull-through was associated with a more extended hospital stay. The duration of surgery did not differ significantly between the two techniques [28]. 

Different surgical techniques for Hirschsprung’s disease have variable functional outcomes. Another method, laparoscopy-assisted TEPT (LEPT, LTAPT), has been shown to reduce the incidence of faecal soiling, faecal incontinence, and constipation compared to solely open surgery [35]. This could be attributed to the maintenance of the sphincter’s integrity during laparoscopic dissection (due to the shorter duration of retractor use [36]). A randomised controlled trial of 52 patients who underwent TEPT found that 24 of them had internal anal sphincter (IAS) defects on endosonographic examination, which were more frequent after transanal procedures (69%) than transabdominal ones [22,36]. Postoperative complications due to rectal mobilisation and anal sphincter stretching were also more common after TEPT than after the Duhamel pull-through [37]. To prevent postoperative constipation, some strategies include keeping the rectal cuff short after TEPT and leaving the native rectum short after the Duhamel pull-through [38]. 

A large meta-analysis comparing TEPT with the open Duhamel technique, the laparoscopic-assisted Duhamel technique, and LEPT in 4781 patients—of whom 2039 underwent TEPT—suggested that LEPT might be the superior procedure. TEPT was associated with more significant blood loss during surgery, longer recovery time for gastrointestinal function, more extended hospital stays, and higher rates of complications, such as HAEC [28]. However, another study found no significant differences between LEPT and TEPT regarding incontinence, stricture, constipation, enterocolitis, and soiling. TEPT was reported to have faster recovery of normal bowel function, while LEPT had slightly fewer postoperative complications with similar long-term functional outcomes [39]. LEPT for rectosigmoid HD was shown to be a safe procedure, with almost 90% of patients achieving normal bowel function by puberty [40]. 

Laparoscopy-assisted pull-through procedures have the advantage of preserving the marginal arteries and veins during colon mobilisation, which may improve the vascularisation of the distal pull-through colon. In contrast, transanal procedures without laparoscopic assistance often compromise the marginal arteries to the pull-through colon, resulting in less optimal blood supply. Different diathermy techniques, such as LigaSure and hook diathermy, are safe and effective for tissue sealing during laparoscopic-assisted colorectal dissection, with no significant differences in postoperative complications, such as bleeding, leakage, perianal excoriation, or enterocolitis [41]. Moreover, indocyanine green fluorescence angiography (ICG-FA) may help assess tissue perfusion and identify patients at risk of complications due to inadequate vascular supply. ICG-FA could potentially enhance the evaluation of rectal pull-throughs and optimise patient outcomes [42].

## 5. Implications for Future Research and Clinical Practice

Based on our clinical experience, the future research should focus on identifying risk factors that predispose patients to complications following TEPT. Large-scale, multicentre studies are needed to validate the findings and provide a more comprehensive understanding of the factors that influence outcomes. Additionally, there is a need for studies exploring alternative surgical techniques or modifications of the current approach. Clinically, our findings suggest the need for a tailored approach to the management of HD, particularly in patients at higher risk of complications. Enhanced postoperative care protocols, including regular monitoring of signs of enterocolitis and faecal incontinence, are crucial for improving long-term outcomes. Finally, patient education regarding potential risks and the need for regular evaluation is essential for optimising patient care and expectations.

## 6. Conclusions 

The transanal endorectal pull-through (TEPT) procedure has proven to be a viable and effective surgical approach for treating Hirschsprung’s disease in paediatric patients, offering the benefits of a minimally invasive technique. Despite the associated challenges, including a notable incidence of postoperative complications such as enterocolitis and faecal incontinence, the procedure demonstrates satisfactory long-term outcomes when managed by experienced surgeons. The findings underscore the necessity for meticulous patient selection, preoperative planning, and postoperative care for mitigating complications. Ongoing research and refinement of surgical techniques are essential for enhancing the efficacy and safety of TEPT further, ensuring improved quality of life for patients with Hirschsprung’s disease.

## Figures and Tables

**Table 1:** The table summarises the inclusion and exclusion criteria for this study.

Inclusion Criteria	Exclusion Criteria
Children diagnosed with Hirschsprung’s disease who underwent single-stage TEPT between January 2005 and December 2023	Patients who underwent multi-stage surgical procedures.
Diagnosis confirmed by clinical, radiological, and histopathological findings.	Incomplete medical records.
Patients with complete medical records and follow-up data.	Patients lost to follow-up within six months post-surgery.

**Table 2:** Retrospective study: summary of collected data.

Demographics	Preoperative Assessment	Surgery	Postop.	Follow-Up
Age	Symptoms	Date	Hospital stays	Duration
Gender	Presentation	Resected length	Stooling	Stool frequency
Weight	Radiology	Operative time	Complications	Soiling/constipation
	Histopathology	Complications		Enterocolitis

**Table 3:** Complications in similar retrospective studies.

Complication	Samujh et al. [31]	Szymczak et al. [32]	Prytula et al. [33]	Beltman et al. [34]	Current Investigation
**Postoperative** **enterocolitis**	11.3%	8.5%	10.53%	15%	26%
**Anastomotic** **leakage**	4.3%	1.4%	-	11%	4%
**Anastomotic** **stenosis**	4.2%	-	1.91%	9%	14%
**Faecal** **incontinence**	4.2%	12.7%	9.57%	16.2%	18%
**Constipation**	-	1.4%	4.31%	-	18%

## Data Availability

The raw data supporting the conclusions of this article will be made available by the authors on request.
